# Loss of mutual protection between human osteoclasts and chondrocytes in damaged joints initiates osteoclast-mediated cartilage degradation by MMPs

**DOI:** 10.1038/s41598-021-02246-7

**Published:** 2021-11-22

**Authors:** Quitterie C. Larrouture, Adam P. Cribbs, Srinivasa R. Rao, Martin Philpott, Sarah J. Snelling, Helen J. Knowles

**Affiliations:** 1 Department of Pathology, University of Pittsburgh, Pittsburgh, USA; 2 Botnar Research Centre, Nuffield Department of Orthopaedics Rheumatology and Musculoskeletal Sciences, University of Oxford, Headington, Oxford, OX3 7LD UK; 3 Nuffield Department of Surgical Sciences, University of Oxford, Oxford, UK

**Keywords:** Cartilage, Bone, Mechanisms of disease

## Abstract

Osteoclasts are multinucleated, bone-resorbing cells. However, they also digest cartilage during skeletal maintenance, development and in degradative conditions including osteoarthritis, rheumatoid arthritis and primary bone sarcoma. This study explores the mechanisms behind the osteoclast–cartilage interaction. Human osteoclasts differentiated on acellular human cartilage expressed osteoclast marker genes (e.g. *CTSK*, *MMP9*) and proteins (TRAP, VNR), visibly damaged the cartilage surface and released glycosaminoglycan in a contact-dependent manner. Direct co-culture with chondrocytes during differentiation increased large osteoclast formation (*p* < 0.0001) except when co-cultured on dentine, when osteoclast formation was inhibited (*p* = 0.0002). Osteoclasts cultured on dentine inhibited basal cartilage degradation (*p* = 0.012). RNA-seq identified *MMP8* overexpression in osteoclasts differentiated on cartilage versus dentine (8.89-fold, *p* = 0.0133), while *MMP9* was the most highly expressed MMP. Both MMP8 and MMP9 were produced by osteoclasts in osteosarcoma tissue. This study suggests that bone-resident osteoclasts and chondrocytes exert mutually protective effects on their ‘native’ tissue. However, when osteoclasts contact non-native cartilage they cause degradation via MMPs. Understanding the role of osteoclasts in cartilage maintenance and degradation might identify new therapeutic approaches for pathologies characterized by cartilage degeneration.

## Introduction

Osteoclasts are large multinucleated cells that resorb bone. They form by the fusion of CD14 + monocytes and regulate skeletal development and maintenance via homeostatic interactions with bone-forming osteoblasts.

It is often overlooked that osteoclasts also digest cartilage (osteoclasts on cartilage are sometimes termed ‘chondroclasts’^1^). During skeletal development, endochondral ossification replaces cartilage templates with mineralized bone following osteoclast-mediated digestion of the calcified cartilage matrix^2–4^. Osteoclasts also degrade the calcified cartilaginous callus during bone fracture healing. Mice deficient in osteoprotegerin (OPG), a negative regulator of osteoclasts, degrade the cartilaginous callus faster, exhibit increased osteoclast numbers and faster union of the fractured bone^5^.

Dysregulation of the balance between osteoclasts and osteoblasts towards relative osteoclast overactivation causes pathological osteolysis in conditions including osteoporosis, bone cancer and rheumatoid arthritis (RA). Cartilage destruction within affected joints is characteristic of RA. Inflammatory cytokines activate fibroblast-like synoviocytes and T cells to produce osteoclastogenic macrophage colony stimulating factor (M-CSF) and receptor activator of nuclear factor kappa B ligand (RANKL), promoting monocyte / macrophage fusion to form multinucleated osteoclasts. These osteoclasts drive joint destruction via physical contact with bone and both non-mineralised and calcified cartilage^6,7^. Osteoclasts also invade articular cartilage in human knee osteoarthritis (OA)^8^, the most common disease of cartilage loss.

Inflammatory cytokines such as TNFα potentiate the osteoclastogenic effect of RANKL^9^, potentially explaining how TNFα-blockade protects against bone and cartilage loss in patients with RA^10^. In human TNF-transgenic mice, which develop destructive arthritis associated with enhanced osteoclast formation, both an anti-TNFα antibody and the osteoclast-inhibiting bisphosphonate zoledronate block bone erosion and cartilage damage and reduce osteoclast formation within the inflamed synovium^11^. Denosumab, a monoclonal anti-RANKL antibody, inhibits osteoclast formation, suppresses bone resorption and causes a reduction in the cartilage turnover in patients with RA^12^. Osteoclast-inhibiting bisphosphonates^13,14^ and OPG^15^ are chondroprotective in murine models of OA.

Mature osteoclasts have a morphology highly specialised for bone-resorption^16^. Resorption is preceded by osteoclast attachment to the bone matrix, mediated by integrins such as the vitronectin receptor (VNR, αvβ3 integrin). This is followed by polarization and cytoskeletal reorganization; F-actin-rich podosomes form a dynamic actin ring which, alongside the integrins, forms a sealing zone isolating the highly folded bone-facing ruffled border membrane from the extracellular environment^16^. Ultrastructural features of osteoclasts resorbing calcified cartilage exhibit features common to bone-resident osteoclasts including abundant mitochondria, vacuolation, lysosomes and deep infoldings at points of contact with the calcified matrix^4^.

Osteoclasts on bone and those on cartilage also have similar basic molecular characteristics. Multinucleated cells form within the inflammatory synovium in RA but only express the calcitonin receptor, a marker of fully differentiated osteoclasts, when they directly contact calcified cartilage or subchondral bone, suggesting that contact with mineralised tissue directs the final stages of differentiation^17^. Multinucleated cells digesting cartilage in RA, OA and the primary bone tumour giant cell tumour of bone (GCTB) express an osteoclast phenotype (CD14 − , HLA-DR − , CD45 + , CD68 + CD51 + , TRAP + , cathepsin K + and MMP9 +)^18^.

However, the mechanisms of both osteoclast formation on cartilage and osteoclast degradation of cartilage are poorly understood. Osteoblasts produce the necessary cytokines for homing osteoclastogenesis to bone and chondrocytes can also produce these factors^19,20^. CD14 + monocytes differentiated on healthy human articular cartilage express immunophenotypic markers characteristic of osteoclasts (CD68 + , CD14 − and CD51 +) and degrade cartilage via release of glycosaminoglycans (GAG). Primary human osteoclasts from GCTB and pigmented villonodular synovitis (PVNS) tissue can also release GAG from cartilage^18^. Maintenance of articular cartilage is a balance between anabolic and catabolic pathways with degradation mediated in large part by matrix metalloproteinases (MMPs), which are also produced by osteoclasts^8,21^.

Despite evidence of physical interaction between osteoclasts and cartilage in RA, OA and GCTB the mechanism(s) by which osteoclasts degrade cartilage remain largely unknown. This study aimed to enhance our understanding of the interaction between osteoclasts and cartilage; to ascertain whether osteoclasts degrade cartilage using the same cellular machinery as for bone resorption and whether chondrocytes affect this process. If osteoclasts contribute to driving cartilage degradation, understanding and controlling their activity represents an important perspective for diseases characterized by degeneration of cartilage.

## Results

### Osteoclasts can differentiate on cartilage and degrade the cartilage matrix

**Figure 1:**
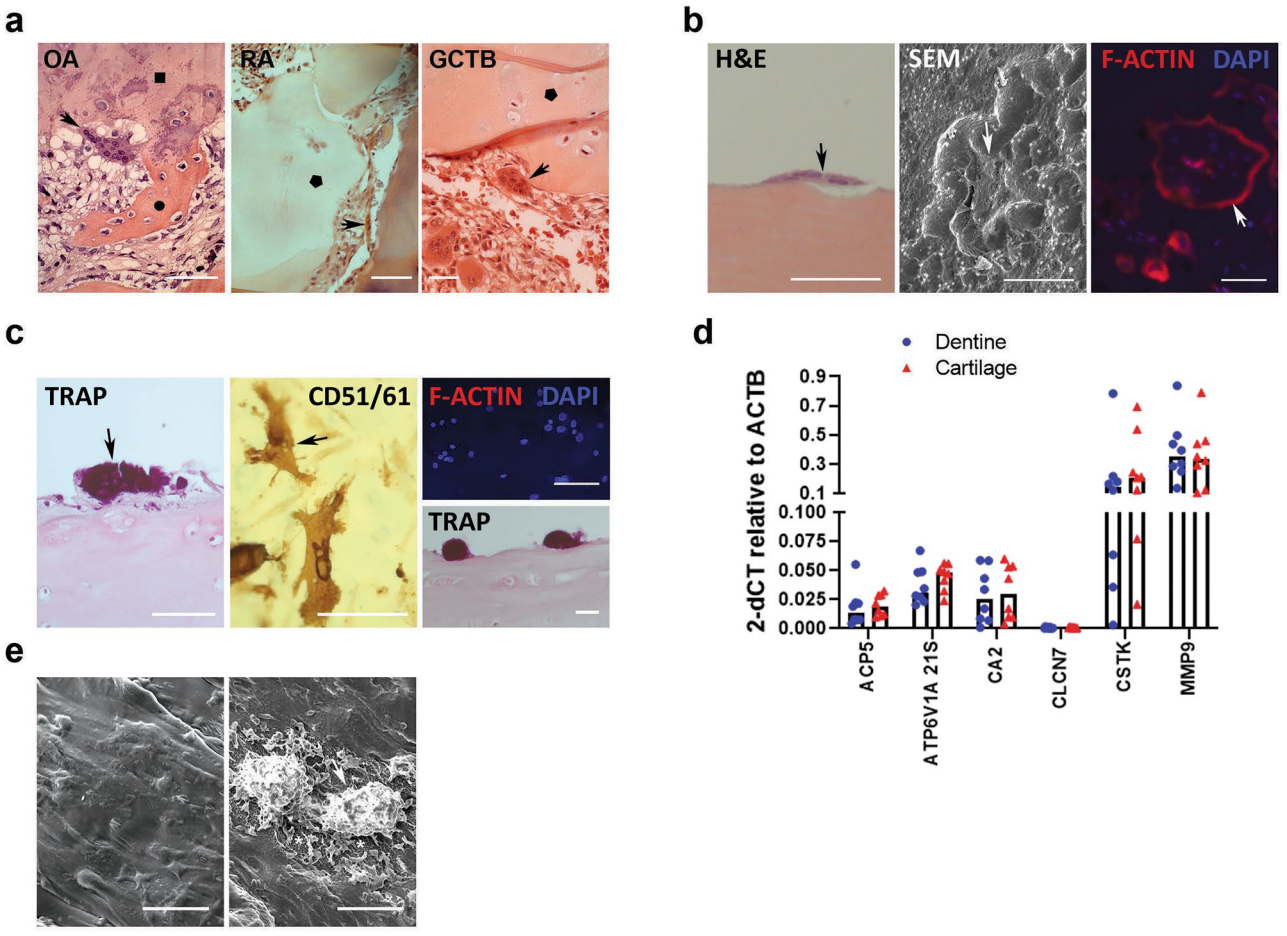
Osteoclasts visibly degrade cartilage matrix. (**a**) H&E staining of OA knee, RA and GCTB tissue. Multi-nucleated osteoclasts (arrow) resorb subchondral bone () and invade mineralised () and unmineralised cartilage (). (**b**) Osteoclasts (arrow) differentiated on dentine with (left) H&E-stained transverse section showing visible subcellular resorption, (middle) SEM image of a 97.49 µm diameter osteoclast surrounded by resorption tracks, and (right) F-actin rings (red) around multi-nucleated (blue) osteoclast. (**c**) Osteoclasts (arrow) differentiated on acellular cartilage showing (left, bottom right) TRAP-stained transverse section through multi-nucleated osteoclasts with no visible subcellular resorption and (middle) CD51/61-positive osteoclasts with (top right) no evidence of F-actin ring formation. (**d**) Expression of classical osteoclast marker genes by osteoclasts differentiated on dentine or acellular cartilage. *ACP5* = *TRAP*, *CLCN7* = H + /Cl − transporter channel 7), *CTSK* = cathepsin K (n = 8; multiple Mann–Whitney). (**e**) SEM images of (left) acellular cartilage and (right) osteoclasts (arrow) on acellular cartilage showing visible degradation the surrounding cartilage matrix (*). All scale bars = 100 μm.

Multinucleated cells morphologically characteristic of osteoclasts were observed adjacent to visibly eroded mineralised and non-mineralised cartilage in cases of OA, RA and GCTB (Fig. 1a). Human osteoclasts differentiated on dentine produced clear resorption pits beneath the cells, visible by both light microscopy and SEM, associated with formation of an F-actin ring (Fig. 1b). In contrast, and despite formation of large multinucleated cells expressing the osteoclast markers TRAP and VNR, human osteoclasts differentiated on unmineralised acellular articular cartilage did not form an F-actin ring and did not perform visible erosion by light microscopy (Fig. 1c). No difference in the expression of classical osteoclast marker genes (*ACP5* (*TRAP), ATP6V1A 21S, CA2, CLCN7, CTSK, MMP9*) was evident between osteoclasts formed on dentine or on cartilage (Fig. 1d). SEM images confirmed that osteoclasts on cartilage were of similar diameter to those on dentine but were more spherical in appearance. The cartilage surface was visibly damaged around osteoclasts despite no visible resorption tracks (Fig. 1e) indicating that osteoclasts do degrade cartilage but, despite having a similar molecular profile, not in the same way as bone matrix.

### Chondrocyte regulation of osteoclast differentiation and function depends on the osteoclast substrate

**Figure 2:**
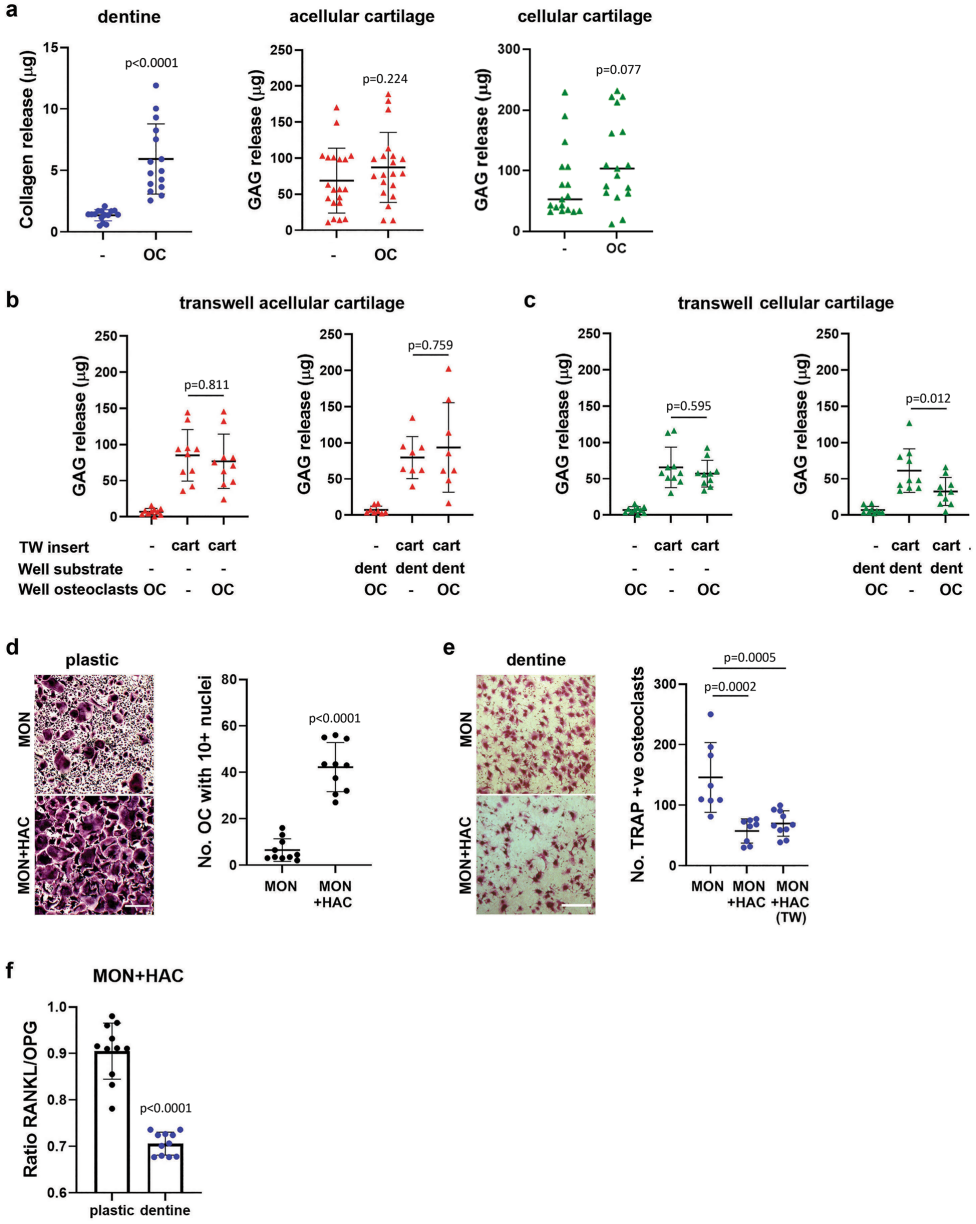
Substrate-dependent regulation of osteoclasts by chondrocytes. (**a**) Osteoclast-mediated release of (left, n = 16; T test) collagen from dentine and release of GAG from (middle, n = 20; T test) acellular or (right, n = 17; Mann–Whitney) cellular cartilage after 13 days of differentiation. (**b**,**c**) GAG release from indirect co-culture experiments with osteoclasts differentiated on either plastic or dentine for 13 days with pieces of (**b**) acellular cartilage or (**c**) cellular cartilage in a Transwell insert. (n = 10; both one-way ANOVA) (**d**,**e**) TRAP-staining following 10 days’ differentiation of CD14 + monocytes into osteoclasts by direct co-culture with chondrocytes and exogenous M-CSF and RANKL. Differentiation performed on (**d**) cell culture plastic (scale bar = 400 µm) with quantification of the number of TRAP-positive cells with ≥ 10 nuclei (n = 10; T test) or (**e**) dentine discs (scale bar = 500 mm) with quantification of the total number of TRAP-positive cells (n = 8; one-way ANOVA). (**f**) Expression ratio of RANKL:OPG in direct co-cultures of osteoclasts and chondrocytes on plastic or dentine (n = 11; T test).

To quantify resorption of different substrates, we measured osteoclast-mediated release of collagen and GAG. Generation of active osteoclasts was confirmed by release of collagen from dentine during resorption pit formation (Fig. 2a). Osteoclasts did not release collagen from cartilage (Supplementary Fig. S1a, b) but showed a non-significant trend to release GAG from both acellular cartilage (chondrocytes killed by freeze-thawing, Supplementary Fig. S1c) and cellular cartilage containing live chondrocytes (Fig. 2a). No increase in GAG release was evident when osteoclasts were cultured on dentine or plastic with the cartilage in a Transwell insert, suggesting that direct contact between osteoclasts and cartilage is required for proteoglycan degradation (Fig. 2b,c). Interestingly, basal GAG release from cellular cartilage was inhibited when distant osteoclasts (those not in direct contact with cartilage) were cultured on dentine but not plastic (Fig. 2c), suggesting a protective influence of bone-resident osteoclasts on the cartilage matrix via effects of secreted products on active chondrocytes.

Direct co-culture of CD14 + monocytes with chondrocytes increased the number of large osteoclasts (> 10 nuclei) formed on plastic (Fig. 2d), but reduced the number formed on dentine in a manner independent of direct contact (Fig. 2e). This might be partially due to a reduced RANKL:OPG expression ratio in chondrocytes cultured on dentine (Fig. 2f). This suggests that factors secreted by chondrocytes inhibit the formation and activity of bone-resident osteoclasts, but that chondrocytes can stimulate osteoclast formation at non-bone sites.

### MMPs drive osteoclast-mediated degradation of cartilage

**Figure 3:**
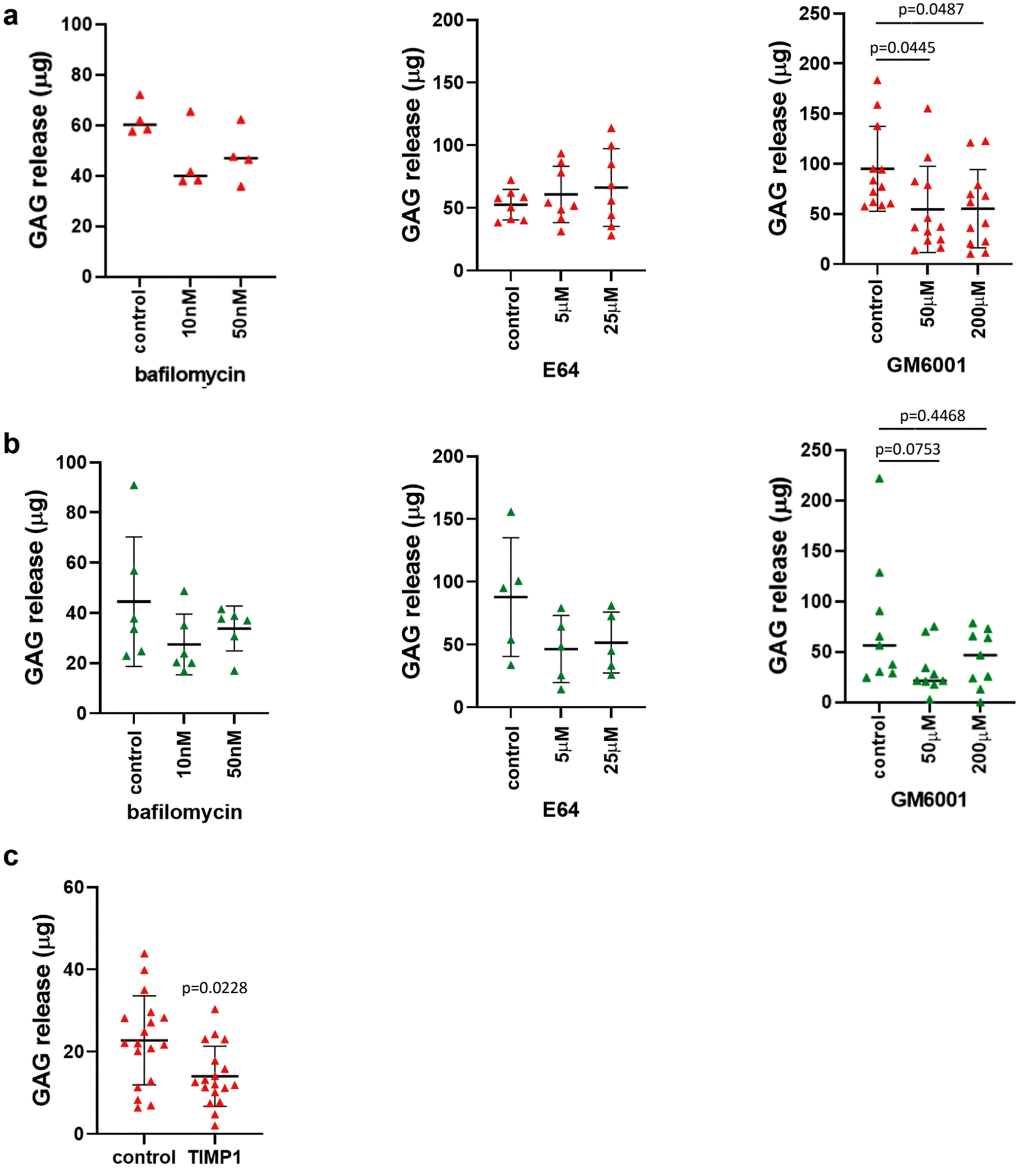
MMPs drive osteoclast-mediated cartilage degradation. (**a**,**b**) Effect of bafilomycin, E64 and GM6001 on osteoclast-mediated release of GAG from (**a**) acellular or (**b**) cellular cartilage after 13 days of differentiation. N varies for each graph, as represented by the number of data points. Statistics: (**a**): bafilomyin, Kruskal–Wallis ANOVA; E64 and GM6001, one-way ANOVA. (**b**) bafilomycin and E64, one-way ANOVA; GM6001, Kruskal–Wallis ANOVA. (**c**) Effect of recombinant human TIMP1 (100 nM) on GAG release from osteoclasts cultured on acellular cartilage (n = 18; T test).

We next sought to identify the proteinases responsible for osteoclast-driven degradation of cartilage by inhibiting key bone resorption components. The efficacy of bafilomycin and E64 to inhibit acidification of the resorption lacunae and cathepsin K activity respectively was confirmed in osteoclasts cultured on dentine (Supplementary Fig. S2a). Neither inhibitor affected osteoclast-mediated release of GAG from cartilage. However, the pan-MMP inhibitor GM6001 significantly reduced osteoclast-mediated GAG release from acellular cartilage (Fig. 3a), while trending towards the same effect in cellular (Fig. 3b) cartilage.

Some of the GM6001-mediated reduction in GAG release in cellular cartilage was due to inhibition of basal release of GAG by GM6001 in the absence of other stimuli (Supplementary Fig. S2b), confirming that live chondrocytes release MMPs that degrade cartilage. Recombinant human tissue inhibitor of metalloproteinase 1 (TIMP1) also inhibited osteoclast-mediated release of GAG from acellular cartilage (Fig. 3c), establishing the active enzyme as a soluble MMP.

### MMP8 and MMP9 drive osteoclast-mediated release of GAG from cartilage

**Figure 4:**
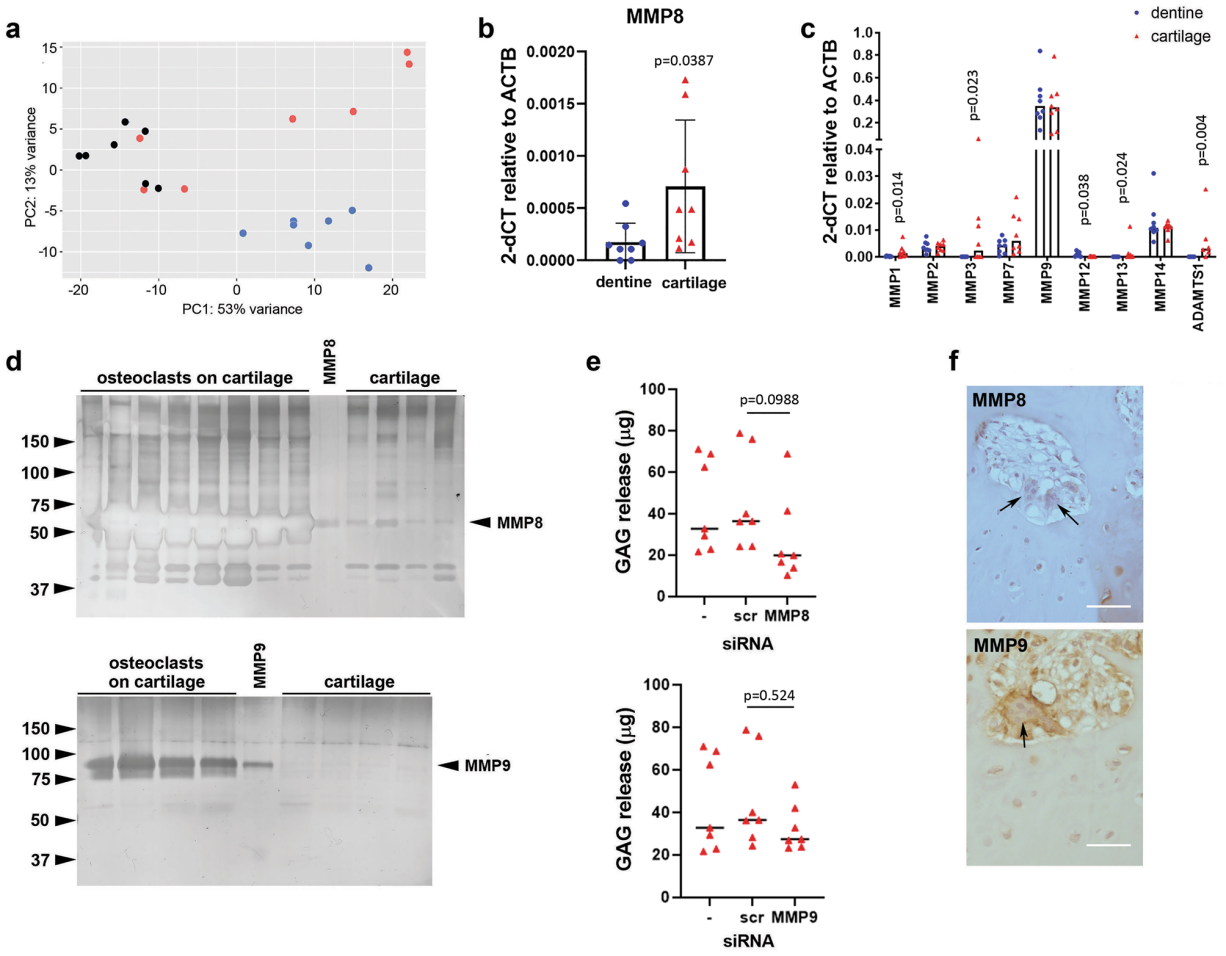
MMP8 and MMP9 drive osteoclast-mediated release of GAG from cartilage. (**a**) Principal Component Analysis (PCA) plot of osteoclasts on acellular cartilage (red), dentine (blue) and cell culture plastic (black), n = 7. (**b**,**c**) RT-qPCR analysis of differences in expression of MMPs by osteoclasts differentiated on dentine or acellular cartilage: (**b**) MMP8 (n = 8; T test); (**c**) MMP1, 2, 3, 7, 9, 12, 13, 14 and ADAMTS1 (n = 8; multiple Mann–Whitney). (**d**) Representative zymography of MMP8 and MMP9 from the conditioned media of osteoclasts differentiated on acellular cartilage compared to recombinant protein and cartilage alone. (**e**) GAG released by osteoclasts differentiated on acellular cartilage and transfected with siRNA targeting MMP8 or MMP9 on day 7 of differentiation, versus no siRNA (−) and scrambled siRNA (scr) controls (n = 7; Kruskal–Wallis ANOVA). (**f**) Human OA tissue sections stained for MMP8 (top) and MMP9 (bottom). Arrows indicate multinucleated osteoclasts. Scale bar = 100 µm.

To identify specific MMPs responsible for osteoclast-mediated cartilage degradation, as well as other contributory matrix-degrading genes, we used RNAseq to compare the expression profile of osteoclasts differentiated on cell culture plastic, acellular cartilage and dentine. Osteoclasts on dentine clustered away from the other substrates, suggesting distinct gene expression profiles between bone-resident osteoclasts and those on ‘non-bone’ substrates (Fig. 4a). *MMP8* showed the greatest fold upregulation in osteoclasts on cartilage versus dentine (8.89-fold, *p* = 0.0133) (Fig. 4b, Supplementary Fig. S3, Table 1). No other top-regulated gene had functions suggestive of an involvement in cartilage degradation. We therefore explored expression of other members of the large sub-family of soluble MMPs. *MMP1*, *MMP3*, *MMP13* and *ADAMTS1* were upregulated in osteoclasts differentiated on cartilage whereas *MMP12* was downregulated. *MMP9* mRNA was consistently expressed at a higher level than other *MMPs* (Fig. 4c). Due to high levels of variability in the expression of these *MMP*s in osteoclasts on cartilage (which were therefore not picked up as differentially expressed by RNAseq), we focussed our attention on MMP8 and MMP9.

**Table 1:** Top 15 over-expressed genes in osteoclasts on cartilage. *LFC* Log fold change; *padj* *p* value adjusted.

Gene	LFC	*p*-adj
MMP8	8.89993669	1.33E−02
PER2	5.33387028	7.07E−08
GCK	4.96826831	1.36E−04
DOCK10	2.26797421	5.30E−07
CCR7	2.0880772	3.35E−04
HEBP2	2.00439463	3.19E−05
TPM2	1.8862969	1.65E−04
STAG3	1.54982285	1.44E−02
H2AFY	1.49036968	9.50E−03
OSBPL8	1.27888291	6.49E−05
DIRAS1	1.19834275	2.47E−02
NSD3	1.19698843	1.65E−04
GATS	1.10256273	2.45E−02
CLDND1	1.09619631	7.22E−05
LGI2	1.08712524	3.24E−02

Gelatin zymography confirmed production of active MMP8 and MMP9 by osteoclasts cultured on cartilage (Fig. 4d), running at approximately 65 kDa (pro-MMP8 75 kDa) and 82 kDa (pro-MMP9 92 kDa) respectively as indicated by the bands in the positive control lanes. Isoform-specific siRNA-mediated knockdown of MMP8 or MMP9 (Supplementary Fig. S4a) reduced osteoclast-mediated GAG release from cartilage explants by 39% and 28% respectively (Fig. 4e), although this trend was not significant. GAG release from cartilage alone was unaffected by siRNA (Supplementary Fig. S4b). Immunohistochemistry of human OA tissue confirmed strong expression of MMP8 and MMP9 in osteoclasts located between the articular cartilage and subchondral bone (Fig. 4f). Chondrocytes surrounded by the cartilage ECM expressed lower levels of both MMPs.

## Discussion

Our data suggests MMP8 and MMP9 as contributory MMPs that drive osteoclast-mediated cartilage degradation and suggests a role for osteoclast–chondrocyte interactions in maintaining joint function. We propose that, in the healthy joint, chondrocytes and bone-resident osteoclasts exert distant effects on the formation and activity of the other cell type to maintain joint integrity. When joint architecture is compromised in conditions such as RA and OA, chondrocytes enhance osteoclast formation within cartilage which drives pathological degradation of the cartilage matrix.

Osteoclasts differentiated on cartilage explants formed large multinucleated cells with the immunophenotype and gene expression profile of osteoclasts on dentine. However, osteoclasts on cartilage had a more rounded three-dimensional profile and did not form the F-actin rings characteristic of sealing zone formation. Human osteoclasts on cartilage in RA also form a less visible ruffled border without actin rings^22^. It may be that minerals are necessary to induce sealing zone formation. Murine osteoclasts on glass coverslips half-coated with apatite only form a sealing zone on the mineralized surface^23^. Rabbit osteoclasts form resorption pits on human femoral cortical bone but not on demineralised bone, again suggesting that osteoclasts do not polarise in the absence of minerals^24^. The absence of a ruffled border and sealing zone in osteoclasts on cartilage is probably due to differences in its mechanical, physical and chemical properties compared to dentine and suggests that acidification is not necessary for osteoclasts to degrade unmineralised cartilage.

Despite the absence of resorption pits, cartilage matrix degradation was evident by SEM. Crumbling of cartilage matrix and fragmentation of collagen fibrils adjacent to osteoclasts is also evident in the mandibles of rat foetuses^4^. We did not observe collagen degradation from cartilage by human osteoclasts in vitro; this may be an issue with the sensitivity of the assay used, a species-specific difference or due to additional osteoclast effects present in the in vivo situation. Cartilage matrix degradation was confirmed by the increased release of cartilage matrix GAG in the presence of osteoclasts, as we described previously^18^.

This study suggests contributory MMPs responsible for cartilage degradation by osteoclasts. Osteoclasts cultured on cartilage secreted active MMP8 and MMP9 and siRNA-mediated inhibition of either MMP showed a trend towards reduced GAG release from cartilage by osteoclasts. Lovfall et al.^21^ also described how inhibition of osteoclast bone-resorption pathways affects their ability to degrade cartilage. In agreement with our study, the pan-MMP inhibitor GM6001 inhibited osteoclast-mediated degradation of cartilage, whereas other inhibitors of osteoclast-mediated bone resorption (cathepsin K inhibitors, vacuolar-type H + -ATPase (V-ATPase) inhibitors) had no effect^21,25^. Lack of effect of V-ATPase inhibitors on osteoclast-mediated cartilage degradation supports the hypothesis that acidification is not necessary for osteoclasts to degrade cartilage. Support for this hypothesis also comes from the bone literature; MMP inhibitors only reduce osteoclast-mediated release of collagen from bone when the bone is decalcified^26^.

Expression of MMP8 and MMP9 was observed in osteoclasts in contact with cartilage in OA tissue, as we have previously described for MMP9^18^. Osteoclasts invading the articular cartilage in knee OA also express MMP1, MMP3 and MMP13^8^. We also found these to be upregulated in osteoclasts differentiated on cartilage, suggesting that they might contribute to osteoclast-mediated cartilage degradation and indicating an intriguing avenue for future investigations.

MMP8 was investigated based on its robust overexpression in osteoclasts cultured on cartilage versus dentine. To our knowledge, the gene expression profile of osteoclasts has not previously been compared when they are in contact with their two primary natural substrates. Most osteoclast studies are performed on tissue culture plastic or glass^27^, failing to address the critical role of the substrate in osteoclast differentiation, polarisation and activation. Publications that do study effects of bone on osteoclastogenesis perform comparisons with osteoclasts cultured on synthetic cell culture plastic^28,29^. We found no difference in the expression of classical osteoclast genes on a bone versus cartilage substrate, an observation mirrored in murine bone marrow macrophages (BMM) differentiated into osteoclasts on devitalised mouse bone versus plastic^29^. However, differential expression of non-classical osteoclast genes could explain the different cytoskeletal arrangements. Crotti et al. found that annexin A8 expression increases in murine BMM-derived osteoclasts cultured on bone versus plastic, which regulates cytoskeletal reorganization and F-actin ring formation in osteoclasts on a mineralised matrix^28^. Principal components analysis revealed osteoclasts on plastic to cluster, to some extent, with osteoclasts on cartilage and away from osteoclasts on bone, suggestive of a broad distinction between the gene expression profile of osteoclasts on their native mineralised bone substrate and those in non-bone, unmineralised environments.

The distinction between bone-resident and non-bone-resident osteoclasts becomes evident when considering interactions between chondrocytes and osteoclasts. Bone-resident osteoclasts exerted a protective effect on cellular, but not acellular, cartilage matrix, suggestive of effects of an unidentified osteoclast secreted product(s) on chondrocytes. Similarly, chondrocytes inhibited distant osteoclast formation on bone. However, when osteoclasts were cultured on plastic (a ‘non-bone’ substrate) they did not exert a protective effect on cartilage matrix and chondrocytes promoted their formation. This suggests a potential homeostatic relationship between osteoclasts and chondrocytes that maintains the integrity of the normal joint but drives matrix degradation in disease conditions where joint integrity has been lost.

Insight into this homeostatic relationship can be obtained from OA. As disease progresses, increased diffusion of small molecules between bone and cartilage allows direct crosstalk between cells via mechanisms including penetration of blood vessels and microcracks into the calcified cartilage and increased hydraulic conductance of the articular cartilage and subchondral bone plate^30–32^. Pathways implicated in the crosstalk include the TGF-β/Smad, Wnt/β-catenin, RANK/RANKL/OPG, and MAPK pathways^33^. The RANK/RANKL/OPG pathway is highly relevant to osteoclasts. RANKL expression increases in human OA cartilage compared with normal cartilage, largely due to its expression by hypertrophic OA chondrocytes^34^. In equine OA, the relationship between articular cartilage RANKL expression and osteoclast density is stronger than in the subchondral bone and correlates with microcrack formation, suggesting that cartilage RANKL recruits osteoclasts which drive cartilage degradation^35^. In C57BL/6J mice with surgically-induced OA, exogenous OPG, an osteoclast inhibitor and decoy receptor for RANKL, protects articular cartilage from OA progression and prevents chondrocyte apoptosis^15^. Further evidence that osteoclasts and chondrocytes cooperate to drive OA comes from experiments using osteoclast-targeting bisphosphonates. Alendronate, which inhibits osteoclast-mediated bone resorption, is chondroprotective in murine models of OA, inhibiting vascular invasion of calcified cartilage and increasing cartilage thickness^13,36^.

This study was limited by the high natural inter-individual variation in both the resorption / digestion capacity of primary human osteoclasts and in the basal rate of cartilage degradation between donors (Fig. 2a). Additionally, the current study focussed on a restricted number of candidate genes that drive osteoclast-mediated degradation of cartilage. Future consideration of more genes-of-interest from our RNAseq and RT-PCR data will provide further insight into the mechanisms driving both contact-dependent and contact-independent effects of osteoclasts on cartilage and chondrocytes, as well as shedding light on why the collagen network in cartilage is not affected by osteoclasts. Beyond this, experiments manipulating expression of these genes in relevant in vivo models will provide an interesting and important next step enabling more detailed dissection of the mechanism(s) described.

In summary, our data demonstrates that osteoclasts can perform proteolysis-driven cartilage degradation via MMPs and that MMP8 and MMP9 might be involved in this process. It provides insight into the complexity of the interactions between bone and cartilage; specifically, how interactions between osteoclasts and chondrocytes depend on whether osteoclasts reside on bone or a non-bone substrate. This data is relevant to future research into a variety of pathological joint conditions where understanding and controlling the activity of osteoclasts presents an attractive option for targeting cartilage degradation.

## Methods

### Osteoclast differentiation

Peripheral blood mononuclear cells were isolated from human leucocyte cones (NHS Blood and Transplant, Bristol, UK; anonymous donors) by density gradient centrifugation. CD14 + monocytes were positively selected (MACS CD14 + microbeads; Miltenyi Biotech, Surrey, UK) and seeded at 0.25 × 10^6^ cells/well into 96-well plates containing dentine discs, 1 × 10^6^ cells/well into 48-well plates containing cartilage pieces, or 1 × 10^6^ cells/well into 24-well plates. After overnight adhesion, dentine and cartilage was transferred to new wells. Osteoclast differentiation was induced with 25 ng/ml M-CSF (R&D Systems, Abingdon, UK) and 50 ng/ml RANKL (Peprotech, London, UK) in α-MEM containing 10% FBS, 50 IU/ml penicillin, 50 μg/ml streptomycin sulphate and 2 mM L-glutamine. Differentiation medium was refreshed every 2–3 days for 10–13 days. For inhibitor experiments, mature osteoclasts were treated with bafilomycin (Cell Signalling Technology, Hitchin, UK), E64 (Cambridge Bioscience, Cambridge, UK), GM6001 (Selleckchem, Munich, Germany) or recombinant human TIMP1 (R&D Systems).

### Preparation of dentine and cartilage

Dentine (elephant ivory; HM Revenue & Customs, Heathrow Airport, UK) was prepared by cutting 250 μm transverse wafers using a low-speed saw and diamond-edged blade (Buehler, Coventry, UK) then punching out 4 mm diameter discs. Cartilage tissue was washed and cut into approximately 6 × 6 mm squares. Cellular cartilage pieces (containing live chondrocytes) were maintained in αMEM for 4 days prior to experimental use. To generate acellular cartilage, explants were stored at − 80 °C, thawed, then snap-frozen in liquid nitrogen to kill the chondrocytes. Despite the histological presence of cell remnants in some cartilage lacunae, fluorescence (Ex 560 / Em 590 nm) read after 4 h incubation with 10% Alamar Blue at 37 °C confirmed that chondrocytes in cellular cartilage were metabolically active compared to those in acellular cartilage pieces.

### Histology and immunohistochemistry

Osteoclasts cultured on dentine or cartilage were fixed in 4% formalin. Dentine discs were decalcified in 0.5 M EDTA prior to paraffin-embedding. H&E staining was performed on transverse 5 µm sections. Antigen retrieval of deparaffinised OA tissue sections was performed in hot citric acid solution. Sections were exposed to primary rabbit monoclonal antibodies against MMP-8 (ab81286, 1:1000, Abcam, Cambridge, UK) or MMP-9 (ab73734, 1:1000, Abcam) overnight at 4 °C and staining was visualised with 3,3′-diaminobenzidine (DAB). Image acquisition was performed using a Zeiss AxioImager MI microscope, AxioCam HRC camera and AxioVision software. Osteoclasts in tissue sections were considered as large, multinucleated cells containing ≥ 3 nuclei; a widely accepted identification criterion^18,37,38^.

### Characterisation of osteoclasts

Tartrate-resistant acid phosphatase (TRAP) staining of formalin-fixed osteoclasts used naphthol AS-BI phosphate as a substrate, reacting the product with fast violet B salt at 37˚C for 3 h. TRAP-positive multinucleated cells containing 3 ≥ nuclei were considered osteoclasts. Vitronectin receptor (VNR, CD51/61, αVβ3-integrin) expression was determined by immunohistochemistry using a murine monoclonal antibody targeting CD51/61 (MCA757G; AbD Serotec, Oxford, UK) and visualised with DAB. Immunofluorescent staining of F-actin filaments was performed on formalin-fixed cells for 30 min using TRITC-conjugated phalloidin (Sigma-Aldrich, Poole, UK), mounted with fluoroshield containing DAPI (Sigma)and visualised at 450–480 nm. Image acquisition was performed using a Zeiss AxioImager MI microscope, AxioCam HRC camera and AxioVision software. Quantification was performed using ImageJ software (National Institutes of Health, Bethesda, USA) to count the average number of osteoclasts per field of view. Fields of view were randomly selected using pre-defined and consistent locations within each well, counting 4 fields of view at 4 × magnification per well with triplicate wells per experimental condition. To avoid observer bias, counting was performed while blinded to the image identity.

### RT-PCR

RNA was extracted in TRIzol (Invitrogen), purified (Direct-Zol RNA miniprep kit; Zymo Research, Cambridge, UK) and reverse transcribed (High capacity cDNA reverse transcription kit; Applied Biosystems, California, USA). Quantitative PCR used Fast SYBR Green Master Mix in a Viia7 Real-Time PCR system (Applied Biosystems, Warrington, UK). Human primers were pre-validated Quantitect primers (*MMP1* (Hs_MMP1_1_SG) , *MMP3* (Hs_MMP3_1_SG), *MMP9* (Hs_MMP9_1_SG) , *MMP13* (Hs_MMP13_1_SG) or *ACTB* (Hs_ACTB_2_SG); Qiagen, Manchester, UK), Primerdesign primers (*MMP8* (JN215643), *ADAMTS9* (JN171532); Primerdesign, Chandlers Ford, UK) or designed in-house using NCBI Primer-Blast (Supplementary Table 1. In-house primers were validated by gradient PCR to determine the optimum annealing temperature, then by RT-PCR melt curve analysis to check for primer dimers and a single amplification product. Validated primers produced a single, sharply defined band of the correct size on an agarose gel). Genes were not considered to be expressed at an average cycle threshold (Ct) value over 34. Expression of individual mRNAs was calculated relative to expression of β-actin (*ACTB*) using the (2-ΔCt) method.

### Scanning electron microscopy

Dentine slices and cartilage pieces were fixed in 4% formalin, sputter-coated with gold using the SC7620 Mini Sputter Coater System (Quorum Technologies, Lewes, UK) and imaged using a Philips XL 30/SEM with field emission gun.

### GAG and collagen release

The 1,9-dimethylmethylene blue (DMMB) assay quantifies sulphated GAGs (chondroitin 4- and 6-sulphate, heparin, keratin and dermatan sulphates). Conditioned media was diluted in NaH_2_PO_4_ / Na_2_HPO_4_ buffer solution (pH 6.5). Diluted sample (40 µl) was mixed with 250 µl DMMB solution (16 mg DMMB, 50 mM glycine, 40 mM NaCl, 0.1 M HCl, pH 3) and the absorbance measured at 530 nm against a chondroitin sulphate standard curve.

Collagen was quantified by measuring hydroxyproline using the dimethylaminobenzaldehyde (DAB) and chloramine T assay. Conditioned media was hydrolysed with an equal volume of concentrated HCl for 18 h at 105˚C. Dried precipitate was resuspended in dH_2_O and 40 μl reacted with 25 μl chloramine T solution (140 mg chloramine T, 2 ml dH_2_O, 8 ml acetate-citrate buffer) for 4 min to oxidise free hydroxyproline. This was incubated with 150 µl DAB solution (30 ml propan-2-ol, 10 ml DAB solution [20 g DAB, 30 ml 70% perchloric acid]) at 65˚C for 35 min. Absorbance was measured at 560 nm and compared with a hydroxyproline standard curve.

### Isolation of chondrocytes

Chondrocytes were isolated from cartilage tissue by overnight digestion with collagenase (1 mg/ml) and passed through a 70 μm cell strainer (Falcon Becton Dickinson, Oxford, UK). Chondrocytes were grown to 80% confluence and used for experiments up to passage 3.

### Co-culture experiments

Direct co-culture was performed with CD14 + monocytes (1 × 10^6^/well) and chondrocytes (4 × 10^4^/well) in 24-well plates. For Transwell experiments, chondrocytes were seeded onto 0.4 μm filter cell culture inserts for 24 h then moved into wells in which CD14 + monocytes had been seeded on cell culture plastic or dentine discs in the lower chamber. For Transwell cartilage experiments, cartilage was placed in the cell culture insert and monocytes in the lower chamber. Cultures were treated for 9–10 days with M-CSF and RANKL as for osteoclast differentiation.

### RNAseq

RNA was DNase I-treated, cleaned and concentrated (Zymo RNA Clean and Concentrator, Zymo Research) then enriched for poly(A) mRNA (NEBNext poly(A) mRNA Magnetic Isolation Module, NEB Biosystems, Ipswich, UK). Sequencing libraries were prepared using the NEBNext Ultra II RNA Library Prep kit (New England Biolabs). RNA and DNA quality were assessed using High Sensitivity RNA or DNA Screentape and an Agilent 4200 tapestation. Single-indexed and multiplexed samples were run on an Illumina Next Seq 500 sequencer using a NextSeq 500 v2 kit (FC-404–2005; Illumina, Can Diago, CA) for paired-end sequencing.

A computational pipeline was written calling scripts from the CGAT toolkit (https://github.com/cgat-developers/cgat-flow)^39,40^. Sequencing reads were de-multiplexed based on the sample index and aligned to the human genome assembly version 38 (GRCh38) using the STAR (Spliced Transcripts Alignment to a Reference) aligner^41^. At least 14 million aligned reads were obtained per sample. Reads were mapped to genes using featureCounts v1.4.6 (part of the subreads package), in which only uniquely mapped reads were counted to genes. Differential expression analysis was performed using DESeq2^42^ between three groups: osteoclasts cultured on cell culture plastic, dentine or cellular cartilage.

### Gelatin zymography

On day 9 of differentiation, media was changed to FBS-free media containing M-CSF and RANKL. Conditioned media was collected after 24 h, centrifuged, mixed with 2 volumes of Zymogram sample buffer (Bio-Rad) and loaded onto a 10% acrylamide gel containing co-polymerised gelatin (ZY00100BOX; ThermoFisher Scientific). Active recombinant human MMP8 and MMP9 (R&D systems) were used as positive controls. Gels were washed in renaturation buffer (BioRad) and then in development buffer (50 mM Tris, 10 mM CaCl2, 50 mM NaCl, 0.05% Brij 35, pH 7.6) to activate MMPs. Gels were processed for silver staining and scanned using an Epson V700 Photo scanner and Epsonscan software.

### MMP8 and MMP9 siRNA

Osteoclasts were differentiated for 7 days before transfection with 25 nM of two separate siRNAs targeting *MMP8* (105332, 112911; Life Technologies), *MMP9* (104072, 113182; Life Technologies) or a non-specific siRNA control using lipofectamine RNAiMax. Duplexes were removed after 6 h and replaced with osteoclast differentiation medium.

### Statistics

Statistical analysis was performed in GraphPad Prism 8 (GraphPad Software, California, USA). D'Agistono Pearson or Shapiro–Wilk were used to test for normality, depending on the sample size. Statistical significance of the unpaired conditions was determined using one-way ANOVA with Tukey’s multiple comparison, Kruskal–Wallis ANOVA with Dunn’s multiple comparison, Mann–Whitney test or *t*-test as appropriate. The False Discovery Rate (FDR) method was used when calculating multiple Mann–Whitney tests, with the two-stage Benjamini, Krieger, & Yekutieli procedure for controlling the FDR ie correcting for multiple testing. Results were considered significant at *p* < 0.05. All figures include at least four independent experiments performed with osteoclasts from different donors (n ≥ 4, each n being the average of 3 technical replicates). Each donor is represented by an individual point on a graph with an indication of either mean ± SD (normally distributed data) or the median (data that is not normally distributed).

### Ethics

Use of leucocyte cones was approved by the London-Fulham Research Ethics Committee (11/H0711/7). Archival pathological bone and joint specimens were obtained from the Nuffield Orthopaedic Centre, Oxford, UK. Cartilage was obtained at the time of surgery from patients undergoing total knee arthroplasty for OA at the Nuffield Orthopaedic Centre. Samples were obtained via the Oxford Musculoskeletal Biobank and collected with informed donor consent in full compliance with national and institutional ethical requirements, the United Kingdom Human Tissue Act and the Declaration of Helsinki (HTA Licence 12217, Oxford REC C 09/H0606/11+5, London-Fulham Research Ethics Committee 07/H0706/81).

## Supplementary Information

Supplementary Information.

## Data Availability

The datasets generated and analysed during the current study are available in the GEO repository (GSE166535) or can be obtained from the authors by reasonable request.

## References

[1] Odgren PR, Witwicka H, Reyes-Gutierrez P (2016). The cast of clasts: Catabolism and vascular invasion during bone growth, repair, and disease by osteoclasts, chondroclasts, and septoclasts. Connect Tissue Res..

[2] Staines KA, Pollard AS, McGonnell IM, Farquharson C, Pitsillides AA (2013). Cartilage to bone transitions in health and disease. J. Endocrinol..

[3] Schenk RK, Spiro D, Wiener J (1967). Cartilage resorption in the tibial epiphyseal plate of growing rats. J. Cell Biol..

[4] Savostin-Asling I, Asling CW (1975). Transmission and scanning electron microscope studies of calcified cartilage resorption. Anat. Rec..

[5] Ota N (2009). Accelerated cartilage resorption by chondroclasts during bone fracture healing in osteoprotegerin-deficient mice. Endocrinology.

[6] Bromley M, Woolley DE (1984). Chondroclasts and osteoclasts at subchondral sites of erosion in the rheumatoid joint. Arthritis Rheum..

[7] Bromley M, Bertfield H, Evanson JM, Woolley DE (1985). Bidirectional erosion of cartilage in the rheumatoid knee joint. Ann. Rheum. Dis..

[8] Shibakawa A (2005). The role of subchondral bone resorption pits in osteoarthritis: MMP production by cells derived from bone marrow. Osteoarthritis Cartilage.

[9] Luo G, Li F, Li X, Wang ZG, Zhang B (2018). TNFalpha and RANKL promote osteoclastogenesis by upregulating RANK via the NFkappaB pathway. Mol. Med. Rep..

[10] Lin YJ, Anzaghe M, Schulke S (2020). Update on the pathomechanism, diagnosis, and treatment options for rheumatoid arthritis. Cells.

[11] Herrak P (2004). Zoledronic acid protects against local and systemic bone loss in tumor necrosis factor-mediated arthritis. Arthritis Rheum..

[12] Cohen SB (2008). Denosumab treatment effects on structural damage, bone mineral density, and bone turnover in rheumatoid arthritis: a twelve-month, multicenter, randomized, double-blind, placebo-controlled, phase II clinical trial. Arthritis Rheum..

[13] Hayami T (2004). The role of subchondral bone remodeling in osteoarthritis: reduction of cartilage degeneration and prevention of osteophyte formation by alendronate in the rat anterior cruciate ligament transection model. Arthritis Rheum..

[14] Kadri A (2010). Inhibition of bone resorption blunts osteoarthritis in mice with high bone remodelling. Ann. Rheum. Dis..

[15] Shimizu S (2007). Prevention of cartilage destruction with intraarticular osteoclastogenesis inhibitory factor/osteoprotegerin in a murine model of osteoarthritis. Arthritis Rheum..

[16] Blangy A (2020). The osteoclast cytoskeleton—current understanding and therapeutic perspectives for osteoporosis. J. Cell Sci..

[17] Gravallese EM (1998). Identification of cell types responsible for bone resorption in rheumatoid arthritis and juvenile rheumatoid arthritis. Am. J. Pathol..

[18] Knowles HJ (2012). Chondroclasts are mature osteoclasts which are capable of cartilage matrix resorption. Virchows Arch..

[19] Komuro H (2001). The osteoprotegerin/receptor activator of nuclear factor kappaB/receptor activator of nuclear factor kappaB ligand system in cartilage. Arthritis Rheum..

[20] Nakao K (2005). Collaborative action of M-CSF and CTGF/CCN2 in articular chondrocytes: possible regenerative roles in articular cartilage metabolism. Bone.

[21] Lofvall H (2018). Osteoclasts degrade bone and cartilage knee joint compartments through different resorption processes. Arthritis Res. Ther..

[22] Bromley M, Woolley DE (1984). Histopathology of the rheumatoid lesion. Identification of cell types at sites of cartilage erosion. Arthritis Rheum..

[23] Saltel F, Destaing O, Bard F, Eichert D, Jurdic P (2004). Apatite-mediated actin dynamics in resorbing osteoclasts. Mol. Biol. Cell.

[24] Chambers TJ, Thomson BM, Fuller K (1984). Effect of substrate composition on bone resorption by rabbit osteoclasts. J. Cell Sci..

[25] Neutzsky-Wulff AV (2010). Alterations in osteoclast function and phenotype induced by different inhibitors of bone resorption–implications for osteoclast quality. BMC Musculoskelet. Disord..

[26] Henriksen K (2006). Degradation of the organic phase of bone by osteoclasts: A secondary role for lysosomal acidification. J. Bone Miner. Res..

[27] Sharma SM (2006). Genetics and genomics of osteoclast differentiation: Integrating cell signaling pathways and gene networks. Crit. Rev. Eukaryot. Gene Expr..

[28] Crotti TN (2011). Bone matrix regulates osteoclast differentiation and annexin A8 gene expression. J. Cell Physiol..

[29] Purdue PE (2014). Comprehensive profiling analysis of actively resorbing osteoclasts identifies critical signaling pathways regulated by bone substrate. Sci. Rep..

[30] Hwang J (2008). Increased hydraulic conductance of human articular cartilage and subchondral bone plate with progression of osteoarthritis. Arthritis Rheum..

[31] Pan J (2012). Elevated cross-talk between subchondral bone and cartilage in osteoarthritic joints. Bone.

[32] Botter SM (2011). Osteoarthritis induction leads to early and temporal subchondral plate porosity in the tibial plateau of mice: an in vivo microfocal computed tomography study. Arthritis Rheum..

[33] Zhou X, Cao H, Yuan Y, Wu W (2020). Biochemical signals mediate the crosstalk between cartilage and bone in osteoarthritis. Biomed. Res. Int..

[34] Kwan Tat S (2009). Modulation of OPG, RANK and RANKL by human chondrocytes and their implication during osteoarthritis. Rheumatology.

[35] Bertuglia A (2016). Osteoclasts are recruited to the subchondral bone in naturally occurring post-traumatic equine carpal osteoarthritis and may contribute to cartilage degradation. Osteoarthritis Cartilage.

[36] Bikle DD (1994). Alendronate increases skeletal mass of growing rats during unloading by inhibiting resorption of calcified cartilage. J. Bone Miner. Res..

[37] Mahoney DJ (2011). TSG-6 inhibits osteoclast activity via an autocrine mechanism and is functionally synergistic with osteoprotegerin. Arthritis Rheum..

[38] Oshita K (2011). Human mesenchymal stem cells inhibit osteoclastogenesis through osteoprotegerin production. Arthritis Rheum..

[39] Cribbs AP (2019). CGAT-core: a python framework for building scalable, reproducible computational biology workflows. F100 Res..

[40] Sims D (2014). CGAT: Computational genomics analysis toolkit. Bioinformatics.

[41] Dobin A (2013). STAR: Ultrafast universal RNA-seq aligner. Bioinformatics.

[42] Love MI, Huber W, Anders S (2014). Moderated estimation of fold change and dispersion for RNA-seq data with DESeq2. Genome Biol.

