# Supplementary material for: Loss of mutual protection between human osteoclasts and chondrocytes in damaged joints initiates osteoclast-mediated cartilage degradation by MMPs
Source: Sci Rep. 2021 Nov 22;11:22708. doi: 10.1038/s41598-021-02246-7 (PMC8608887; doi:10.1038/s41598-021-02246-7)
Supplement: Supplementary file 1 — Supplementary Information. [file 41598_2021_2246_MOESM1_ESM.pdf]

Supplementary Information for:

Loss of mutual protection between human osteoclasts and chondrocytes in damaged joints initiates osteoclast-mediated cartilage degradation by MMPs

Quitterie C Larrouture[1], Adam P Cribbs[2], Srinivasa R Rao[2,3], Martin Philpott[2], Sarah J Snelling[2], Helen J Knowles[2]*

Affiliations: [1]Department of Pathology, University of Pittsburgh, Pittsburgh, USA. [2]Botnar Research Centre, Nuffield Department of Orthopaedics Rheumatology and Musculoskeletal Sciences, University of Oxford, UK. [3]Nuffield Department of Surgical Sciences, University of Oxford, Oxford, UK.

Supplementary Table 1: RT-PCR primer sequences designed in-house.

Gene name	Forward primer	Reverse primer
ACP5	TGAGGACGTATTCTCTGACCG	CACATTGGTCTGTGGGATCTTG
ATP6V1A 21S	CCATCGGAACTACCATGCAGG	TCCACAGAAGAGGTTAGACAGG
CA2	GGACAAAGCAGCTTCACTTGG	CCTAGAACGGCCAGTCCATC
CLCN7	CTAAGAAGGTGTCCTGGTCCG	GGAGGGTCCATATCCTGGCG
CTSK	GCAGAAGAACCGGGGTATTGA	GAAGGAGGTCAGGCTTGCAT
MMP2	TACAGGATCATTGGCTACACACC	GGTCACATCGCTCCAGACT
MMP7	GAGTGAGCTACAGTGGGAACA	CTATGACGCGGGAGTTTAACAT
MMP12	AACCAACGCTTGCCAAATCC	TTTCCCACGGTAGTGACAGC
MMP14	ATCTGCCTCTGCCTCACCTA	AAGCCCCATCCAAGGCTAAC

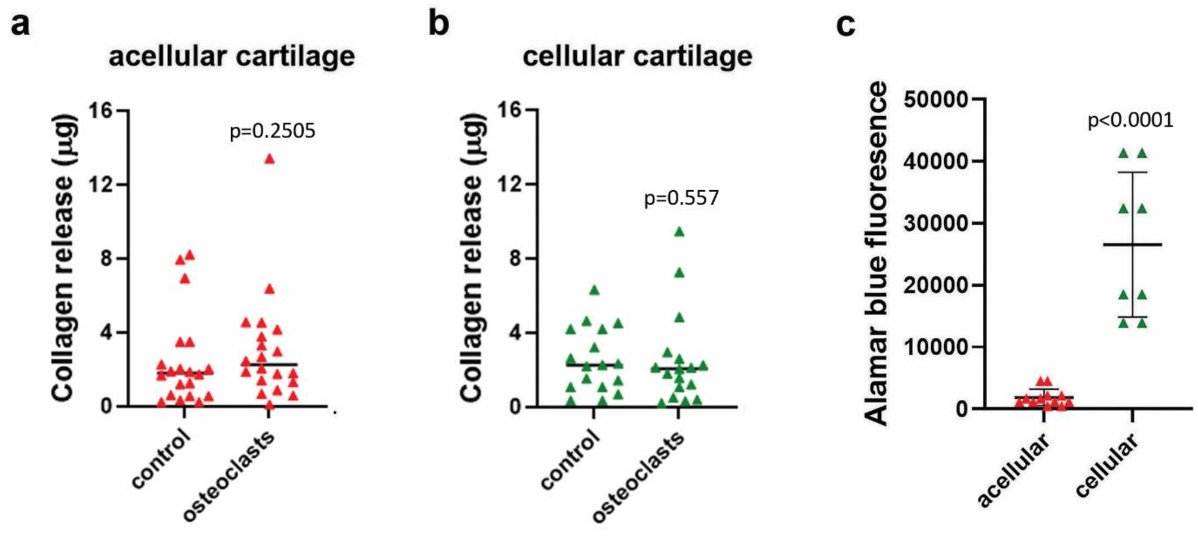

Supplementary Figure 1: Substrate-dependent regulation of osteoclasts by chondrocytes. (a, b) Osteoclast-mediated release of collagen from (a) acellular (n=20; Mann-Whitney) or (b) cellular (n=17; Mann-Whitney) cartilage after 13 days of differentiation. (c) Alamar blue fluorescence of acellular and cellular cartilage on day 13 of culture (no osteoclasts, n=8; T test).

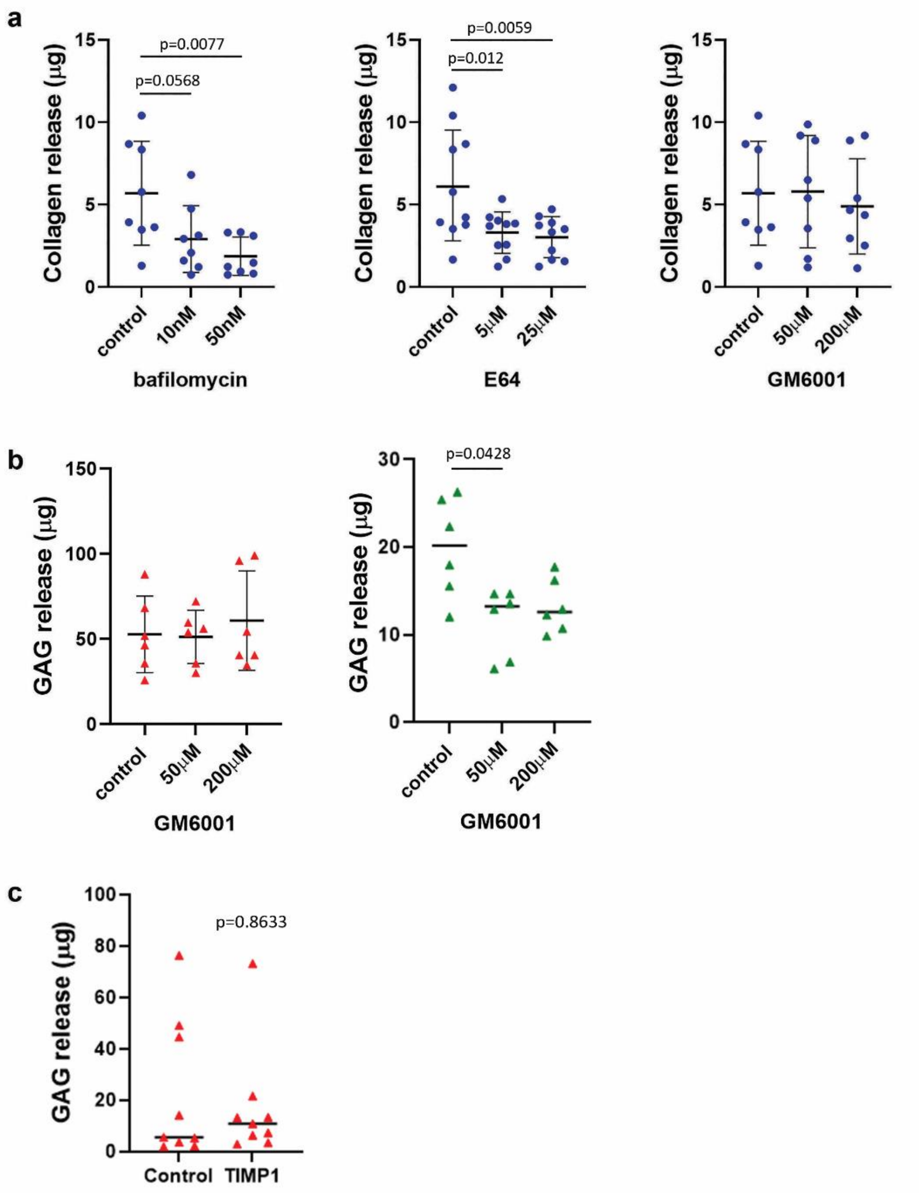

Supplementary Figure 2: MMPs drive osteoclast-mediated degradation of cartilage. (a) Effect of bafilomycin (n=8), E64 (n=10) and GM6001 (n=8) on osteoclast-mediated release of collagen from dentine after 13 days of differentiation. All one-way ANOVA. (b) Effect of GM6001 on basal release of GAG from acellular (red; one-way ANOVA) and cellular (green; Kruskal-Wallis ANOVA) cartilage in the absence of osteoclasts, n=6. (c) Effect of recombinant human TIMP1 (100 nm) on basal release of GAG from acellular cartilage in the absence of osteoclasts (n=9; Mann-Whitney).

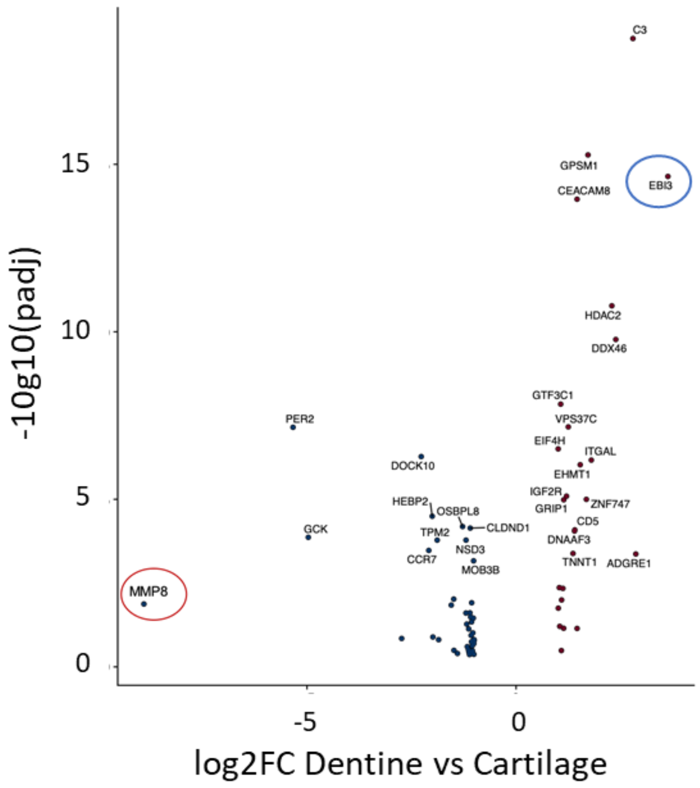

Supplementary Figure 3: Volcano plot showing genes overexpressed in osteoclasts cultured on dentine versus cartilage. Genes overexpressed on cartilage are on the left of the plot, those overexpressed on dentine are on the right. FC, fold change; padj, p value adjusted.

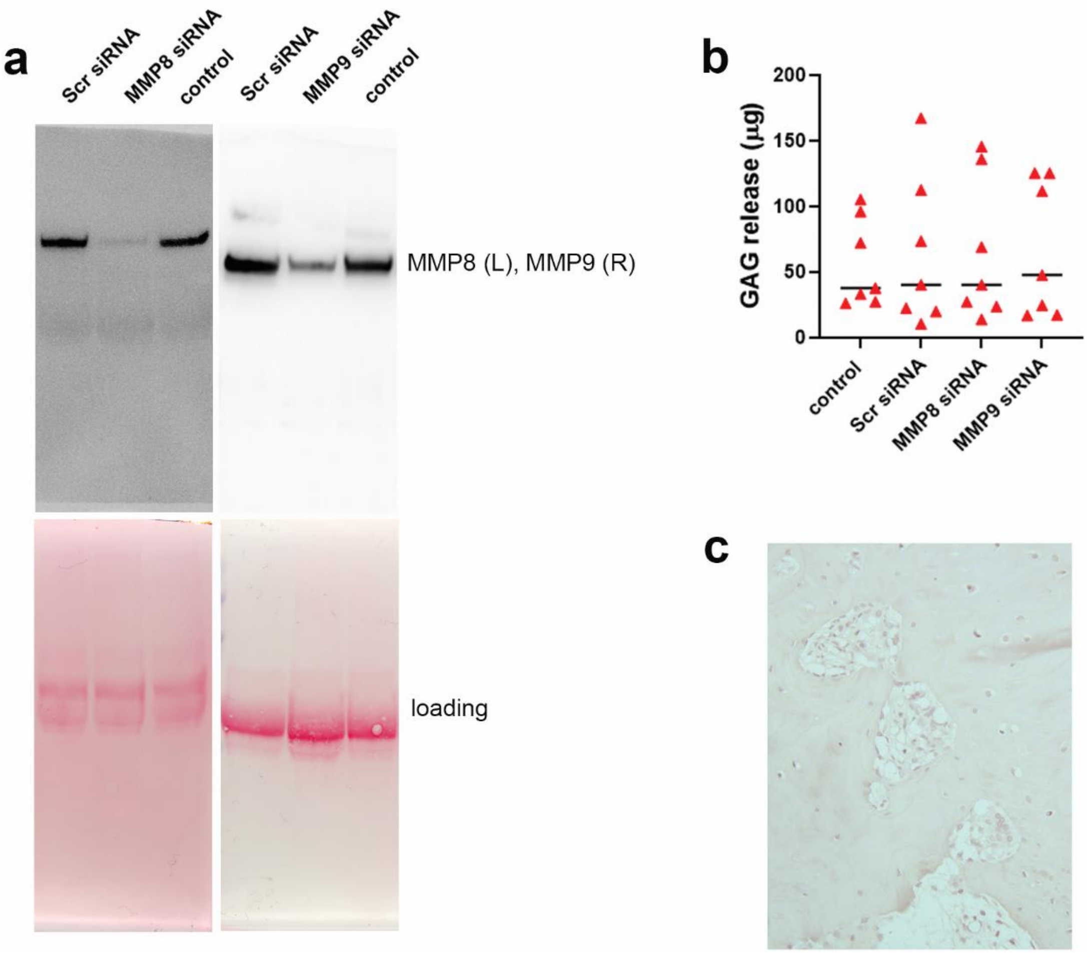

Supplementary Figure 4: MMP8 and MMP9 are the primary MMPs responsible for osteoclast-mediated release of GAG from cartilage. (a) Western blots showing MMP8 and MMP9 expression in osteoclasts following treatment with MMP8- or MMP9-siRNA or a scrambled siRNA control. Loading control = Ponceau staining. (b) Effect of MMP8 or MMP9 siRNA on basal release of GAG from acellular cartilage in the absence of osteoclasts (n=7; Kruskal-Wallis ANOVA). (c) IgG negative control immunohistochemistry of OA tissue section.
